# Host Modulation in Periodontology: Redefining Therapy Beyond Scaling and Root Planing

**DOI:** 10.7759/cureus.93880

**Published:** 2025-10-05

**Authors:** Pankaj Dewanjee, Anootpal Gogoi, Shubham K Srivastava, Chinmoy Sikdar, Akshim Rana

**Affiliations:** 1 Department of Periodontology, Regional Dental College, Guwahati, IND; 2 Department of Prosthodontics, Sardar Patel Post Graduate Institute of Dental and Medical Sciences, Lucknow, IND

**Keywords:** anti-inflammatory agents, dental scaling, host-pathogen interactions, periodontal diseases, root planing

## Abstract

The management of periodontal diseases has traditionally centered on mechanical debridement through scaling and root planing, a cornerstone of periodontal therapy. While effective in reducing bacterial load and disrupting biofilm, this approach alone does not fully address the complex host-mediated inflammatory pathways that drive disease progression and tissue destruction. Advances in molecular biology and immunology have revealed the critical role of host response modulation in periodontal pathogenesis, leading to the development of adjunctive therapeutic strategies that go beyond mechanical intervention. Emerging agents, such as subantimicrobial dose doxycycline, non-steroidal anti-inflammatory drugs, pro-resolving lipid mediators, and host-targeted biologics, demonstrate potential in attenuating destructive inflammation while promoting resolution and regeneration. This editorial explores the evolving concept of host modulation in periodontology, emphasizing its role in redefining therapeutic goals, improving long-term outcomes, and personalizing care for patients with periodontitis. By integrating host-modulatory approaches into routine practice, periodontology is poised to transition from a solely pathogen-centered model to a comprehensive, host-focused paradigm.

## Editorial

Periodontal disease has long been regarded as a chronic inflammatory condition initiated by pathogenic bacterial biofilm and perpetuated by the host immune response. For decades, the clinical management of periodontitis has relied predominantly on mechanical debridement through scaling and root planing (SRP). This cornerstone therapy effectively removes supra- and subgingival biofilm and calculus, thereby reducing microbial challenge and improving clinical parameters such as probing depth and bleeding on probing. However, SRP alone does not entirely halt disease progression in all patients, particularly those with systemic comorbidities, genetic predisposition, or an exaggerated inflammatory response. It has become increasingly clear that while the bacterial biofilm is the initiator, the dysregulated host response is the primary driver of periodontal tissue destruction [1]. This recognition has stimulated a shift from a pathogen-focused treatment model to one that incorporates host modulation as an integral component of comprehensive periodontal therapy.

The host response in periodontitis is multifaceted, involving innate immunity, adaptive immunity, inflammatory mediators, and tissue remodeling pathways. Bacterial lipopolysaccharides and virulence factors trigger immune cells to release cytokines such as interleukin-1β, tumor necrosis factor-α, and prostaglandin E2, which amplify inflammation and activate matrix metalloproteinases (MMPs). These MMPs, in turn, degrade extracellular matrix components, leading to clinical attachment loss and alveolar bone resorption. Furthermore, persistent inflammation impairs the resolution phase, preventing effective tissue healing and regeneration. Thus, the host’s exaggerated and unresolved inflammatory reaction is central to the pathogenesis of periodontitis. Traditional therapies primarily aimed at bacterial reduction leave this destructive cascade relatively unchecked. Consequently, even after meticulous debridement, residual inflammation may persist, and susceptibility to disease recurrence remains high [1].

Host modulation therapy (HMT) was conceptualized to address this therapeutic gap by targeting the dysregulated host response rather than solely eliminating the microbial trigger. The goal of HMT is not to suppress immunity entirely, which would compromise defense against pathogens, but rather to modulate the inflammatory response to limit tissue destruction while enhancing mechanisms of repair and regeneration. This nuanced approach recognizes that inflammation is both a protective and potentially destructive process, requiring careful modulation rather than blanket inhibition [1,2]. Over the last two decades, a wide spectrum of pharmacologic and biologic agents has been investigated for their potential in host modulation, ranging from systemically administered drugs to locally delivered molecules and biologics.

One of the most extensively studied agents in this regard is subantimicrobial dose doxycycline (SDD). At a dose of 20 mg twice daily, doxycycline exerts negligible antimicrobial action but significantly inhibits MMP activity, thereby reducing collagen breakdown and connective tissue loss. Multiple clinical trials have demonstrated the adjunctive benefit of SDD with SRP in reducing probing depth and improving clinical attachment levels, particularly in patients with refractory periodontitis [1,2]. Beyond MMP inhibition, SDD also possesses anti-inflammatory and antioxidant properties, making it a cornerstone in the clinical application of HMT. Notably, in subjects with type 2 diabetes mellitus, a group with heightened susceptibility to periodontitis, SDD treatment has been shown to significantly reduce MMP-9 and MMP-13 levels in gingival crevicular fluid, underscoring its role in mitigating host-derived tissue destruction in high-risk populations [3]. Despite its effectiveness, long-term use requires careful patient selection, monitoring for gastrointestinal tolerance, and consideration of compliance.

Non-steroidal anti-inflammatory drugs (NSAIDs) have also been explored as host modulators due to their ability to inhibit cyclooxygenase enzymes and reduce prostaglandin E2 levels, which are elevated in periodontitis and strongly implicated in bone resorption. Early studies demonstrated that NSAID administration, particularly flurbiprofen and indomethacin, could reduce alveolar bone loss [1]. However, their widespread use has been limited by concerns over systemic side effects, such as gastrointestinal ulceration and cardiovascular risks, especially with long-term use. As a result, NSAIDs have not achieved routine clinical use in periodontology, but their study paved the way for exploring safer alternatives that target similar inflammatory pathways [1,2].

More recently, attention has turned toward pro-resolving mediators, such as lipoxins, resolvins, and protectins, which are derived from polyunsaturated fatty acids. Unlike conventional anti-inflammatory drugs that block inflammatory pathways, these specialized pro-resolving mediators (SPMs) actively promote the resolution of inflammation and return tissues to homeostasis [4,5]. Experimental models have demonstrated that SPMs not only reduce leukocyte infiltration and cytokine release but also stimulate tissue regeneration and bone preservation [4,5]. Although clinical translation is still in early stages, SPMs represent a promising frontier in periodontal host modulation, aligning with the biological need to resolve rather than merely suppress inflammation.

Antioxidants have also emerged as potential host modulators, given that oxidative stress is a key driver of periodontal tissue damage. Agents such as vitamin C, vitamin E, coenzyme Q10, and polyphenols from green tea have been studied as adjuncts in periodontal therapy. By neutralizing reactive oxygen species and improving endothelial function, these antioxidants may complement conventional periodontal treatment [1]. While some studies show modest clinical benefits, the heterogeneity of formulations, dosages, and delivery systems limits definitive conclusions. Nonetheless, the concept of targeting oxidative stress highlights the expanding dimensions of host modulation beyond traditional pharmacologic agents.

Another important dimension of host modulation in periodontology is the use of biologics that specifically target cytokines or signaling pathways involved in periodontal inflammation. Monoclonal antibodies against tumor necrosis factor alpha (TNF-α), interleukin 6 (IL-6), or receptor activator of nuclear factor kappa-B ligand (RANKL), which are already used in conditions such as rheumatoid arthritis, have shown promising effects in preclinical periodontal models [1,2]. Such biologics could theoretically suppress destructive inflammation and bone resorption in severe cases of periodontitis. However, their high cost, systemic side effects, and the chronic nature of periodontitis pose practical challenges to their integration into routine care. Still, as personalized and precision medicine advances, targeted biologics may become part of future periodontal treatment protocols for selected patients with refractory disease [2].

Antimicrobial photodynamic therapy (aPDT) has gained increasing attention as an adjunctive tool, utilizing light-activated photosensitizers to generate reactive oxygen species that not only reduce microbial load but also attenuate pro-inflammatory cytokines and matrix metalloproteinases [6-8]. Clinical studies and meta-analyses have reported improvements in probing depth reduction and clinical attachment gain when aPDT is combined with scaling and root planing, particularly in patients with persistent inflammation or systemic comorbidities such as diabetes mellitus [7-9]. In addition, recent work suggests that aPDT may influence host immune responses by enhancing resolution pathways, thereby offering a dual benefit of microbial control and host modulation [6,9].

Parallel to this, nanotechnology is opening novel avenues for targeted modulation of periodontal inflammation. Nanoparticle-based systems, including liposomes, polymeric nanoparticles, metallic nanoparticles, and nanofibers, are being engineered to deliver anti-inflammatory drugs, antioxidants, and specialized pro-resolving mediators directly into periodontal pockets [10-12]. These nanoscale carriers enhance drug stability and bioavailability, allow for controlled and sustained release, and can be functionalized to selectively target inflamed periodontal tissues [11,12]. Preclinical studies have demonstrated that nanoplatforms can suppress oxidative stress, reduce inflammatory mediator expression, and promote bone regeneration [10,11]. Although translation into routine clinical practice is still in the early stages, such nanotherapeutics represent a promising frontier by aligning precision drug delivery with biologically informed host modulation [10-12].

Host modulation also extends to lifestyle and systemic health interventions. Conditions such as diabetes mellitus, obesity, and smoking are strongly associated with altered host responses and increased periodontal susceptibility [3]. Improving glycemic control, achieving weight reduction, and smoking cessation can all be considered forms of host modulation that enhance the body’s ability to manage periodontal inflammation. Nutritional interventions, physical activity, and stress management may also indirectly modulate host pathways. This broader, patient-centered view aligns with the current emphasis on holistic and interdisciplinary periodontal care, recognizing that local periodontal therapy must be integrated with systemic health optimization.

The clinical integration of HMT requires a balance between efficacy, safety, cost-effectiveness, and patient compliance. At present, subantimicrobial dose doxycycline remains the only host-modulating agent with U.S. Food and Drug Administration (FDA) approval for adjunctive periodontal therapy [1]. Other agents, such as SPMs and biologics, are at various stages of preclinical or clinical investigation [4,5]. The lack of long-term, large-scale randomized controlled trials has limited widespread adoption, and further research is needed to establish optimal dosing regimens, safety profiles, and patient selection criteria [2]. Importantly, clinicians must also be cautious in presenting HMT not as a replacement for conventional mechanical therapy but as a complementary strategy to enhance outcomes, particularly in patients with persistent inflammation despite meticulous debridement.

The future of host modulation in periodontology is intertwined with the broader trend toward precision medicine. Advances in genomics, proteomics, and metabolomics are improving our ability to identify patients who may respond favorably to specific host-modulatory interventions. Biomarkers such as cytokine profiles, MMP levels, and genetic polymorphisms may soon guide tailored therapeutic strategies, allowing clinicians to select the most effective adjunctive therapy for individual patients [2]. This personalized approach could maximize clinical benefits while minimizing risks, ushering in a new era of biologically informed periodontal therapy.

In redefining therapy beyond scaling and root planing, host modulation represents not just a pharmacologic adjunct but a conceptual shift in periodontal care. By recognizing the centrality of the host response in disease progression, periodontology is evolving from a purely pathogen-elimination paradigm to one that embraces biological modulation and resolution of inflammation. This evolution carries profound implications for patient care, clinical outcomes, and the long-term sustainability of periodontal health. While SRP will remain foundational, its synergy with host-modulatory approaches offers a more comprehensive and forward-looking model of therapy. As ongoing research expands the armamentarium of host-modulating agents, clinicians and researchers must work together to translate these advances into practical, evidence-based clinical protocols. Ultimately, the integration of host modulation into routine practice has the potential to transform periodontology into a discipline that not only manages disease but also actively promotes regeneration, resilience, and long-term oral health.
